# State‐of‐the‐Art and Synthetic Challenges for Hydrosilane Production

**DOI:** 10.1002/chem.70978

**Published:** 2026-04-24

**Authors:** Gabriel Durin, Albane Fontaine, Jean‐Claude Berthet, Thibault Cantat

**Affiliations:** ^1^ CEA CNRS, NIMBE Université Paris‐Saclay Gif‐sur‐Yvette France; ^2^ Univ Grenoble Alpes, DCM CNRS Grenoble France

**Keywords:** energy efficiency, H_2_, hydrosilane synthesis, reduction, sustainable chemistry

## Abstract

Hydrosilanes, key compounds in the silicone industry, are mild and selective reducing agents. They have recently proven to be particularly effective in the reductive hydrosilylation of carbon–oxygen bonds of industrial oxygenated wastes (CO_2_, biomass, plastics) for their valorization as carbon resources. However, the current production of hydrosilanes is energy intensive. This review traces the history of hydrosilane preparations from elemental silicon, by electrochemical or metal hydride reduction procedures and summarizes promising new routes to obtain Si─H bonds from H_2_, by hydrogenolysis of (pseudo)halosilanes. The energy efficiencies of these reactions are discussed. The main advances but also the remaining challenges for a virtuous synthesis and recycling of hydrosilanes are highlighted.

## Graphical abstract

The synthesis of hydrosilanes is currently energy intensive and their recycling is not considered. However, these compounds are important in many industrial fields but also for the valorization of oxygenated waste, towards a circular economy. This review compares the available synthetic routes and highlights the remaining challenges for the production of renewable hydrosilanes.

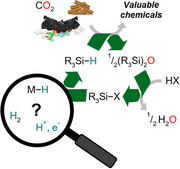

## 1 Introduction

Hydrosilanes R_4−n_SiH_n_ are very important compounds in a number of fields. They are used in the synthesis of high‐purity silicon, a dominant material in the semiconductor industry. Hydrosilanes are subjected to high temperature and converted to pure elemental silicon by chemical vapor deposition [1]. Hydrosilanes are also useful building blocks in the polymer industry. Through dehydrocoupling reactions, Si─Si linkages can be formed to lead to polysilanes of industrial interest, while the Si─C linkages obtained from the hydrosilylation of olefins can modulate the properties of silicone polymers [2]. In recent years, hydrosilanes have attracted increasing interest [3, 4, 5, 6, 7], as mild reducing agents in organic synthesis for instance. Unlike H_2_, they have a weaker polarised bond (BDE(SiH_4_) = 95 kcal.mol^−1^ vs 104 kcal.mol^−1^ for H_2_) [8]. Their redox potential can be modulated by changing the substituents on the Si atom (–0.5/–0.7 V vs. SHE) and these compounds are well suited to reduce C─O bonds (range 0.0/–0.5 V vs. SHE) with high selectivity and under mild conditions [9]. For example, they can reduce amides into amines [10], CO_2_ to formate [11], formaldehyde [12] or methanol [13] derivatives and can promote the reductive depolymerisation of natural and artificial polymers, such as lignin [14], polyethers, polyesters or polycarbonates [15] into valuable products.

Recently, phosphines generated in situ by the reduction of phosphine oxide species with hydrosilanes proved to be excellent catalysts in the reduction of unactivated alkenes to the corresponding alkanes. This P^III^/P^V^ redox cycle process with hydrosilanes also rendered the Wittig reaction catalytic in phosphine for the conversion of a variety of aldehydes and alkyl bromides to the corresponding alkenes [16, 17].

The valorisation of natural raw materials (biomass) and the reprocessing of gaseous and solid anthropogenic effluents such as CO_2_ and some oxygenated plastics is a major environmental challenge. Hydrosilanes, if produced readily, can be useful to reduce these oxygenated materials to recover their carbon content for the production of fuels or high value‐added chemicals.

**SCHEME 1:**
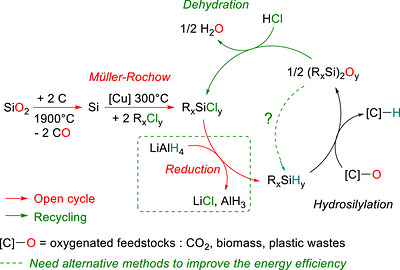
Current (left and down) and alternative (right and up) synthetic routes to hydrosilanes.

One of the main drawback of C–O bond hydrosilylation is the production of siloxane by‐products of little interest that accumulate and can cause pollution (Scheme 1) [18]. Moreover, hydrosilanes are currently synthesised by an energy‐intensive process. In the presence of elemental carbon, silica is first reduced at high temperature to silicon and then transformed into chlorosilanes by the Müller–Rochow process [19]. Finally, the latter are reduced by LiAlH_4_, which is produced from elemental lithium (E° = −3 V vs. SHE), resulting in hydrosilanes. The whole process releases large amounts of CO/CO_2_ and metal chloride salts with a high energy loss as heat. It is therefore interesting to improve the efficiency of the current hydrosilane production process but also to develop recycling pathways.

A new route to hydrosilanes from siloxanes or alkoxysilanes would be of great interest, especially as alkoxysilanes can be easily prepared from silica and alcohols [20]. However, the reduction of these compounds is very difficult because of the high stability of the Si–O bond (BDE Si─O: 190 kcal.mol^−1^) [8]. In this strategy of converting Si─O to Si─H, an additional step might be considered to convert the Si─O bonds into more easily reduced Si─X bonds (X = OTf, I, Br, Cl). While the transformation of siloxanes to silyl triflates [21] or chlorosilanes [22, 23, 24] by treatment with the corresponding acid is known, the final step of converting Si–X bonds is the remaining challenge to overcome, to improve the recyclability but also the overall synthesis of hydrosilanes.

This review presents the state‐of‐the‐art of different synthetic methods to produce hydrosilanes from halosilanes, particularly in the case of organosilanes. These methods are summarised and compared with a focus on the renewable character of each method, especially in terms of energy efficiency.

## 2 The Müller–Rochow Process

**SCHEME 2:**
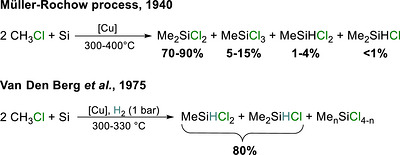
Synthesis of chloro(hydro)silanes by the Müller–Rochow process.

Chlorosilanes were among the first silanes prepared on a laboratory scale and are currently used in industry to prepare organosilicon derivatives, which are important commercial products. The Müller–Rochow process or “direct process” is the common industrial method for the synthesis of organochlorosilanes that was discovered simultaneously in the 1940's by the Rochow group in the United States and the Müller group in Germany [19]. In this process, elemental silicon is reacted with alkyl halides (Cl, Br) in the vapour state (T ≥ 300°C), usually in the presence of a copper catalyst for a better efficiency (Scheme 2). Elemental silicon can be used pure or alloyed with metals that promote its reaction. A complex mixture of products is obtained with some alkyl(halo)silanes. The reaction between methyl chloride and Si(0) has led to the formation of hydrochlorosilanes, such as MeSiHCl_2_ and Me_2_SiHCl. These later compounds are of great industrial interest due to the presence of orthogonal reactive sites, useful in polymerisation or functionalisation reactions, but they are found in minor amounts in the crude mixture.

The successful synthesis of methylchlorosilanes bearing a silicon‐hydrogen bond was achieved by Van Den Berg et al. [25], by the addition of H_2_ to the previous gas phase system. Methyl chloride reacts with Si(0) in presence of a copper powder catalyst, in the temperature range 300°C–330°C under 1 atmosphere of H_2_, to give an overall selectivity of 80% in Me_2_SiHCl and MeSiHCl_2_.

In any case, this process is energy intensive and requires harsh conditions. This synthetic strategy is also limited to the methylchlorosilanes and always needs an additional purification step (often distillation) [19].

## 3 Synthesis of Hydrosilanes by Treatment of Chloro‐ and Alkoxysilanes With Metal Hydrides

### 3.1 Hydrides of the Group I and II Elements

#### 3.1.1 Lithium and Sodium Hydrides

**SCHEME 3:**
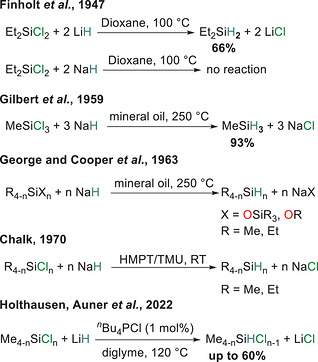
Reduction of chlorosilanes and siloxanes with lithium and sodium hydrides.

The first synthesis of hydrosilanes by reduction of chlorosilanes with alkaline metal hydrides such as LiH or NaH was pioneered by Finholt et al. in 1947 [26]. The reaction is carried out by dropwise addition of chlorosilane in a suspension of lithium hydride in dioxane at 100°C (Scheme 3). Following this procedure, Et_2_SiH_2_ was obtained in 66% yield from Et_2_SiCl_2_. No reaction took place with sodium hydride. Using a gas flow procedure, Gilbert et al. obtained a high yield in MeSiH_3_ (93%) from the reaction of gaseous MeSiCl_3_ passing through a suspension of NaH in mineral oil at 250°C [27]. The scope of hydrosilanes has been considerably extended by George and Cooper et al. who demonstrated that the reduction of alkoxysilanes and even siloxane derivatives was possible with NaH but at a significant energy cost (T >200°C) [28, 29, 30]. Nevertheless, the direct conversion of siloxanes into hydrosilanes is of particular interest for the recyclability and sustainability of hydrosilane synthesis. Alternatively, Chalk produced efficiently the trialkylsilanes R_3_SiH species by treating R_3_SiCl with NaH in phosphoramide or urea solvent at room temperature (RT) [31]. Influence of the solvent, by coordinating on chlorosilane or by increasing the solubility of NaH, may explain this performance. Recently, in 2022, Holthausen and Auner extended the use of LiH for the selective synthesis of chlorohydrosilanes [32]. To perform such reaction, they combined the reduction reaction with a redistribution catalyst to maximise the amount of highly interesting compounds such as Me_2_SiHCl or MeSiHCl_2_. The reaction was carried out in diglyme at 120°C using a phosphonium salt as catalyst and the chlorohydrosilanes were obtained in good yields (up to 60%).

#### 3.1.2 Calcium and Magnesium Hydrides

**SCHEME 4:**
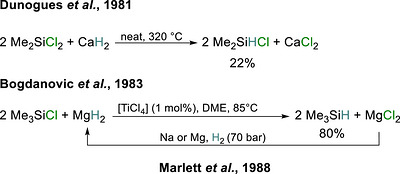
Reduction of chlorosilanes with calcium and magnesium hydrides.

In 1981, Dunogues et al. reported the ability of calcium(II) hydride to reduce Me_2_SiCl_2_ to the very valuable monohydride compound Me_2_SiHCl, under stoichiometric conditions (Scheme 4) [33]. The reaction was very selective, but showed low efficiency and Me_2_SiHCl was obtained in only 22% yield despite the harsh reaction conditions (neat, 320°C). Two years after, Bogdanovic et al. demonstrated that magnesium(II) hydride in the presence of a catalytic amount of TiCl_4_ (1 mol%) generated Me_3_SiH in 80% yield from Me_3_SiCl [34]. The reaction took place under milder conditions (reflux of DME at 85°C). A notable improvement in the latter reaction was reported by Marlett et al. who designed a circular process that regenerate the reductant MgH_2_ from the by‐product MgCl_2_ under H_2_ at 70 bar, in the presence of metallic Na or Mg [35]. In the absence of catalyst, the lower reactivity of these alkaline earth metal(II) hydrides compared to the MH (M = Na, Li) analogues must be compensated by higher temperatures for the reaction to take place.

### 3.2 Hydrides of the Group XIII and XIV Elements

#### 3.2.1 Aluminium Hydrides

**SCHEME 5:**
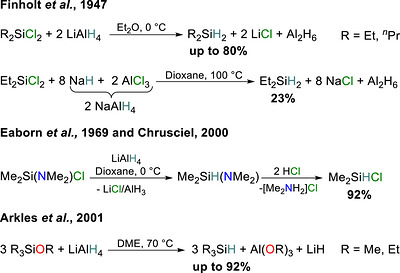
Reduction of chloro‐ and alkoxysilanes with lithium and sodium aluminium hydrides.

Anionic aluminium hydrides are common reagents for lab‐scale synthesis of hydrosilanes and have been widely used in this purpose. In a pioneering study, Finholt et al. highlighted the higher reactivity of LiAlH_4_ compared to LiH by requiring lower temperatures (0 vs. 100°C) for example to convert *^n^* Pr_2_SiCl_2_ to *^n^* Pr_2_SiH_2_ in 80% yield (Scheme 5) [26]. Although competent in a similar reduction, NaAlH_4_ was found to be less reactive, and when prepared *in situ* by combining NaH (6 eq) and AlCl_3_ (3 eq), it reacted with Et_2_SiCl_2_ to give Et_2_SiH_2_ in the highest yield of 23%. Eaborn et al. and Chrusciel developed a two‐step procedure for the selective synthesis of the desired Me_2_SiHCl. It involves the reduction of dimethylaminochlorosilanes to dimethylaminosilanes at 0°C using LiAlH_4_ followed by an acidic treatment with HCl to give Me_2_SiHCl in excellent yield (92%) [36, 37]. Finally, Arkles et al., reported in 2001 the almost quantitative reduction of an alkoxysilane by LiAlH_4_ in dimethoxyethane (DME) [38]. In this case, a higher temperature of 70°C is required. This is consistent with the fact that an alkoxide is a worse leaving group than a chloride, in a reaction that likely involves a SN_2_ mechanism.

#### 3.2.2 Alanes

**SCHEME 6:**
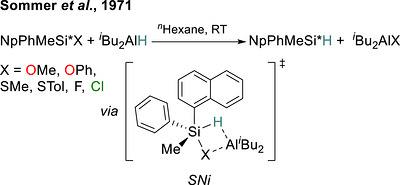
Reduction of chiral silanes with alanes (Np: naphtyl; SNi: internal nucleophilic substitution).

Neutral alanes of formula R_2_AlH (R = alkyl, H) were also tested for the reduction of halosilanes (Scheme 6). Sommer et al. thus reported in 1971 the successful conversion of sulfido‐, alkoxo‐, phenoxo‐, fluoro‐ and chlorosilanes into the corresponding hydrosilanes with the alane *^i^* Bu_2_AlH [39]. A chiral silane was considered to afford stereoselective hydrosilane compounds but no yield was provided. Nonetheless, the group noted that the reactivity of the R_3_SiX species is in the order OR > F >> Cl which is a reverse trend to that observed in the reduction with LiAlH_4_, where alkoxysilanes are more difficult to reduce than chlorosilanes. With the alanes, the reaction would proceed *via* a four centers SNi (internal Nucleophilic Substitution) mechanism and not through the classical SN_2_. This suggestion is related to the retention of configuration observed in the reduction of some chiral silanes. In this case, the reactivity is enhanced because the halosilane and the alane would form an adduct (Scheme 6). The σ donor character of the bridging group X in the adduct would render the aluminium hydride more reactive, thus facilitating the migration of the hydride to the Si atom. Neutral and anionic aluminium hydrides therefore exhibit distinct reactivities and offer alternative ways to the targeted silanes.

#### 3.2.3 Borohydrides

**SCHEME 7:**
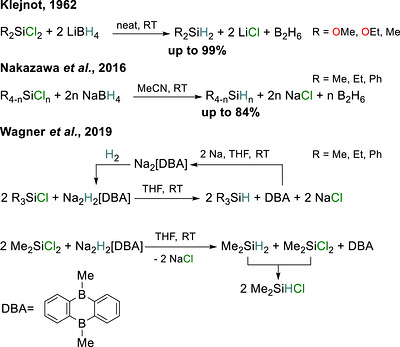
Reduction of chlorosilanes by lithium and sodium borohydrides.

In order to avoid the use of highly reactive aluminium hydrides, borohydrides, which are milder reducing agents, were considered to transform Si─Cl bonds into Si─H. In 1963, Klejnot introduced LiBH_4_ as a reagent for this reaction under neat condition and at room temperature (Scheme 7) [40]. Surprisingly, no reaction took place in the presence of MeSiCl_3_. Nonetheless, high yields of dihydrosilanes (up to 99%) were obtained from a variety of dichlorosilanes. Interestingly, the reaction was compatible with alkoxy groups on silicon. More recently, in 2016, Nakazawa et al. [41] reported an efficient procedure to access hydrosilanes by reduction of a series of organochlorosilanes R_4−n_SiCl_n_ (R = alkyl, aryl) with sodium borohydride (NaBH_4_). The reactions took place rapidly (15 min) at room temperature in MeCN. Several hydrosilanes were obtained in excellent yields (up to 88%). This procedure was found to be very selective with high tolerance for nitriles, esters or alkyl chloride groups which is not possible with aluminium hydrides as reducing agents. The reaction also worked with disilane (ClMe_2_Si)_2_ giving (HMe_2_Si)_2_ in 72% yield.

Three years later, Wagner et al. reported a chemical looping method to produce hydrosilanes by using dianionic diborane species Na_2_[DBA] (DBA = 9,10‐dihydro‐9,10‐diboraanthracene) [42]. In the presence of H_2_, this species is converted to a diborohydride Na_2_[DBA]H_2_ which readily reacts with chlorosilanes to give hydrosilanes together with the diborane DBA. Elemental sodium is then used to reduce the diborane to anionic diborane and thus close the cycle. Interestingly, upon reaction of Me_2_SiCl_2_ with the diborohydride, Me_2_SiH_2_ was initially obtained as the main product, but this later redistributed with the starting Me_2_SiCl_2_ (with time or heat) to give Me_2_SiHCl. This comproportionation to Me_2_SiHCl is likely mediated by the borane Lewis acid released into the medium.

#### 3.2.4 Boranes

**SCHEME 8:**
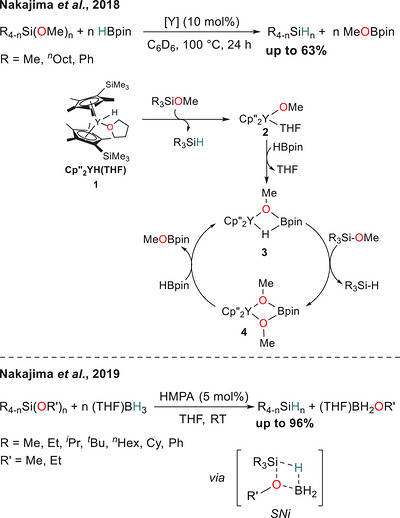
Reduction of alkoxysilanes with boranes catalysed by an yttrium(III) complex or the Lewis base HMPA.

The interest of boranes for the synthesis of hydrosilanes from alkoxysilanes has been highlighted in 2019 by Nakajima et al. (Scheme 8) [43]. The catalytic reduction of R_3_SiOMe (R_3_ = Me_2_Ph, Me_2_(*^n^* Oct), Et_3_) with a mild reductant pinacolborane (HBpin) was performed efficiently with the yttrium(III) metallocene complex (C_5_Me_4_SiMe_3_)_2_YH(THF) (**1**). Despite the high catalytic loading (10 mol%) in **1** and the high temperature required (100°C), the yields in R_3_SiH are moderate (22 to 63%). The steric role of the alkoxy group is demonstrated by the decrease in yield in Me_2_PhSiH when using Me_2_PhSiOR (R = Me, Et, *^i^* Pr), 57, 11 and 0%, respectively. The reduction of the dialkoxysilanes MePhSi(OMe)_2_ and Ph_2_Si(OMe)_2_ also proceeded in moderate yields while that of PhSi(OMe)_3_ led to a mixture where the main hydrosilane PhSiH_3_ is obtained in low yield of 12%. NMR monitoring of the reaction of **1** and Me_2_PhSiOMe showed a slow but quantitative evolution to Me_2_PhSiH and (C_5_Me_4_SiMe_3_)_2_YOMe(THF) (**2**) (within 24 h). Coordination of HBpin on **2** affords the adduct **3** which was isolated in 84% yield. Hydride transfer from **3** to the alkoxysilane with Si─OMe cleavage affords **4** which can readily release MeOBpin.

The same group also reported in 2019 the efficient conversion of a variety of alkoxysilanes using BH_3_ in THF, in the presence of hexamethylphosphoric triamide (HMPA, 5 mol%) as a Lewis base catalyst [44]. The reaction was then studied extensively by considering the cheap and easy‐to‐handle NaBH_4_ in presence of EtBr (as a sacrificial reagent) to generate BH_3_ *in situ*. Again, the transformation of R_3_SiOR to R_3_SiH was found to be sensitive to the nature of the alkoxide. The *tert*‐butyloxide or aryloxide groups did not lead to any reaction. Unfavorable steric hindrance or a poor electron donating OR group thus seems to negatively affect the reaction. These points reflect the importance of the coordination of the alkoxy on the boron atom of BH_3_ for the reaction to occur. A SNi type mechanism *via* a four‐center species is the most plausible pathway in this case. The role of the Lewis base catalyst HMPA is not yet fully elucidated, but assistance in the release of the hydrosilane from the four‐membered intermediate can be proposed.

#### 3.2.5 Stannanes

**SCHEME 9:**
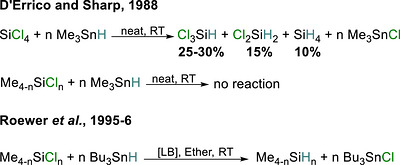
Reduction of chlorosilanes with hydrostannanes. LB = Lewis base.

D'Errico and Sharp introduced the use of trialkylstannane (Me_3_SnH) for the conversion of Si─Cl to Si─H groups (Scheme 9) [45].

The reduction of polyhalosilanes with this reagent proceeds under solvent‐free conditions but suffers from a lack of selectivity. As an example, the three products Cl_3_SiH, Cl_2_SiH_2_ and SiH_4_ were obtained when reacting Bu_3_SnH with SiCl_4_ at room temperature. However, no reaction occurred with alkylchloro‐ or arylchlorosilanes as well as for the reduction of alkylalkoxysilanes.

A decade later, Roewer et al. revisited the transformation of Si─Cl to Si─H using Bu_3_SnH with mono‐ and disilanes (MeSiCl_3_, Me_2_SiCl_2_, Me_3_SiCl, MeCl_2_SiSiMeCl_2_, Me_2_ClSiSiMeCl_2_…), and introduced, for this reaction, a wide range of nitrogen or phosphorus‐based Lewis base catalysts such as a phosphines, phosphites, tertiary amines, imidazole, phosphoramide or N‐heterocycle and phosphonium or ammonium salts [46, 47, 48]. With disilanes, Si–Si bond cleavage can occur if strongly nucleophilic catalysts are employed. Fully reduced silanes (MeSiH_3,_ Me_2_SiH_2_, Me_3_SiH…) were always obtained as the major products from their chloride precursors (MeSiCl_3_, Me_2_SiCl_2_, Me_3_SiCl…) and the emerging trend is that the more chloride the silicon compounds contain, the easier the reaction. Increasing pKa of the base correlates with better yields. The authors then suggested the formation of an adduct between the base and the chlorosilane leading to a hypervalent silicon atom that would react faster than the tetracoordinate silane.

## 4 Synthesis of Hydrosilanes by Reaction of Chloro‐ and Alkoxysilanes With Grignard Reagents

**SCHEME 10:**
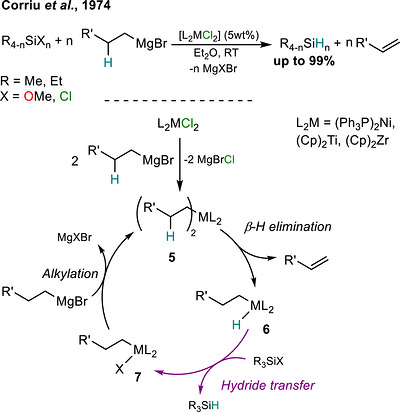
Grignard reagents as reductant for hydrosilane synthesis.

Another way to reduce halo‐ or methoxysilanes involves Grignard reagents (Scheme 10). This method, introduced by Corriu et al. [49, 50, 51], affords two different products, a tetraalkyl/arylsilane or a hydrosilane, depending on whether the alkyl group of the alkyl magnesium halide has a hydrogen atom in the β‐position. The reaction is catalysed by transition metal complexes such as [(PPh_3_)_2_NiCl_2_] or the metallocene [(C_5_H_5_)_2_MCl_2_] (M = Ti, Zr) used in 5 wt%, and proceeds readily at room temperature in Et_2_O. The reductive properties of the *^i^* PrMgBr/[(C_5_H_5_)_2_MCl_2_] system would be similar to those of LiAlH_4_. The scope of the reaction is wide and the conversion of fluoro‐ and chlorosilanes as well as methoxysilanes into hydrosilanes is favoured in high yields (up to 99%). The mechanism proposed by the authors involves, in a first step, the dialkylation of the bis(chloro)precatalyst with formation of the reactive species **5**. This later undergoes facile β‐elimination to form the metal hydride derivative **6**, which readily transfers its hydride ligand to the chlorosilane to form R_3_SiH. A final alkylation step closes the catalytic cycle to regenerate the dialkyl complex **5**.

## 5 Synthesis of Hydrosilanes by Electrochemical Reduction

The growing interest for chloro‐ and hydrosilanes in the 1950's attracted the attention of the electrochemist. Electrochemistry is an interesting strategy for the virtuous synthesis of hydrosilanes from chlorosilanes. Electrons can be produced from renewable energy sources such as solar or wind power and are promising and highly available reducing agents. In addition, the energy consumption for the reaction can be controlled by the applied electrical potential [52].

**SCHEME 11:**
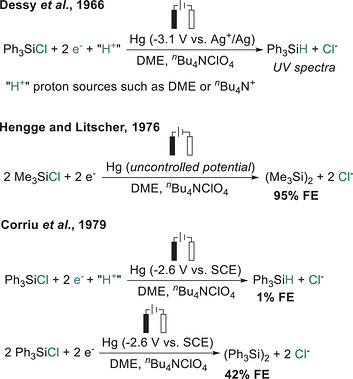
Electroreduction of aryl chlorosilanes in aprotic solvents. FE is the Faradaic Efficiency.

In 1952, Abrahamson et al. first reported the electroreduction of a variety of organochlorosilanes (R_3_SiCl, R_2_SiCl_2_, RSiCl_3_; R = Me, Et, Ph…) [53]. This study was carried out in pyridine with a mercury‐pool cathode to measure the half‐wave potential of some chlorosilanes. However, electrolysis of chlorosilanes was not performed and the possible reduction products remained undetermined. In 1966, an exhaustive electrolysis of the arylchlorosilanes Ph_3_SiCl and Ph_2_SiCl_2_ at a mercury‐pool cathode was carried out in 1,2‐dimethoxyethane (DME) containing *^n^* Bu_4_NClO_4_ as supporting electrolyte by Dessy et al. (Scheme 11) [54]. The authors demonstrated by UV spectroscopy that the solutions after electrolysis are identical to those of authentic Ph_3_SiH and Ph_2_SiH_2_ samples. The formation of the arylhydrosilanes occurred at −3.1 and −1.9 V vs. Ag^+^/Ag (−2.5 and −1.3 V vs. SCE), respectively and the proton source for the formation of hydrosilanes would come from the solvent DME or the *^n^* Bu_4_N^+^ cation. These results contrast with those of Hengge and Litscher, who reported for the electrolysis of Me_3_SiCl the formation of (Me_3_Si)_2_ with 95% FE, under similar conditions but without control of the applied potential [55].

**SCHEME 12:**
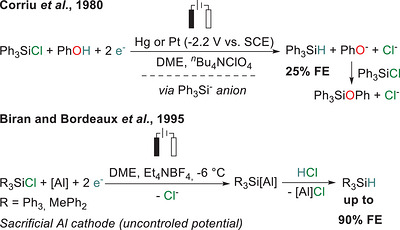
Electroreduction of aryl chlorosilanes with proton sources.

In 1979, Corriu et al. highlighted the importance of anhydrous conditions on the nature of the products of chlorosilanes electrolysis [56]. They pointed out that even a very small amount of water (<0.03%) in the organic solvent can have a huge influence on the reduction current. A solution of Me_3_SiCl in MeCN, dried by standard distillation methods, showed a reduction peak at −0.4 V vs. SCE which corresponds to the reduction of HCl released by the hydrolysis of Me_3_SiCl [54]. Under the same conditions as reported by Dessy et al. but without traces of water [54], electrolysis of Ph_3_SiCl in DME at –2.6 V vs. SCE produced Ph_3_SiH with a faradic efficiency (FE) of 1%. The dimer (Ph_3_Si)_2_ was the main product, obtained in 42% FE. While many studies [57, 58, 59, 60, 61] have confirmed and extended the electrochemical conversion of Si─Cl into Si─Si bonds, only two reports have demonstrated the formation of hydrosilanes by isolating these products (Scheme 12).

In the first report, the electrochemical reduction of R_3_SiX compounds (R = Ph and X = F, Cl, Br; R_3_ = Me_3_, Ph_2_Me, Ph_2_H, PhMeH and X = Cl) was studied by a battery of electrochemical methods (polarography, cyclic voltammetry, controlled potential coulometry and preparative scale electrolysis). The reduction of these silicon compounds exhibits a single irreversible wave. Although disilanes are the main products of the preparative scale electrolysis in aprotic solvents, hydrosilanes dominate in protic solution. Corriu et al. reported that, in the presence of phenol as proton source, Ph_3_SiH is obtained in 25% FE from Ph_3_SiCl with the concomitant formation of the side‐product Ph_3_SiOPh (25%) [62]. The formation of the disilane or the Si−H species would result from two distinct processes. In the former, the disilanes would be obtained by dimerization of the R_3_Si^•^ radical, which originates from a one‐electron reduction. However, at a higher cathodic potential, the reactive anion R_3_Si– generated by two electron transfers reacts with R_3_SiCl to give eitttther (Ph_3_Si)_2_ in aprotic solvents or the hydride R_3_SiH in protic solutions.

The second report, by Biran and Bordeaux et al., involves the electroreduction of arylchlorosilanes (Ph_3_SiCl and Ph_2_MeSiCl) to the corresponding arylhydrosilanes using a sacrificial aluminium cathode (Scheme 12) [63]. During the electrolysis of MePh_2_SiCl, the dimerisation reaction leading to the symmetric disilane was avoided by trapping the derived silyl anion MePh_2_Si^−^ as a silylaluminium intermediate. This latter is stable and was isolated as an oily compound. Reaction of this silylaluminium “MePh_2_Si‐Al” intermediate with HCl gave the desired arylhydrosilane MePh_2_SiH with high FE (up to 90%). It was also reacted with D_2_O to give MePh_2_SiD or with Me_3_SiCl to afford the unsymmetrical disilane MePh_2_SiSiMe_3_ (57%). This electrolysis procedure was also carried out with Ph_3_SiCl and PhMe_2_SiCl and the corresponding hydrosilanes Ph_3_SiH and PhMe_2_SiH were obtained in *ca*. 50% yield.

All these studies demonstrate that chlorosilanes are difficult to reduce and require very negative potentials (≤ –2 V vs. SCE) compared to those associated with the theoretical formation of hydrosilanes (Si(OEt)_4_, H_2_/SiH_4_), which are in the range of *ca*. –0.75 V vs. SCE (*vide infra* Scheme 19). The electroreduction of halosilanes to hydrosilanes requires further studies to determine the influence of proton sources. In addition, the high overpotential observed (> 1.2 V), which is a good thermodynamic indicator to quantify the energy efficiency of a redox reaction, indicates that direct electron transfer between the electrode and the silicon atom of the chlorosilanes is not efficient. This transformation therefore requires the development of appropriate catalysts or mediators in order to proceed efficiently.

## 6 Synthesis of Hydrosilanes by Hydrogenolysis of Halosilanes, Silyltriflates, and Silyl Enol Ethers

### 6.1 From Silyl Enol Ethers

**SCHEME 13:**
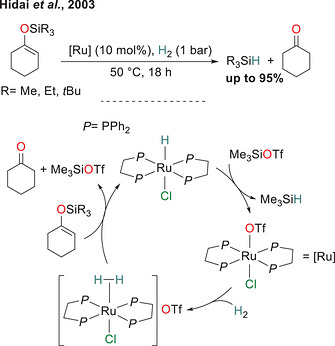
Catalytic hydrogenolysis of silyl enol ethers with a Ru(II) complex. (OTf = CF_3_SO_3_).

A novel catalytic route to hydrosilanes from silyl enol ethers, which uses molecular hydrogen (H_2_) as a reducing agent, was discovered by Hidai et al. in 2003 (Scheme 13) [64]. The catalyst [RuCl(dppe)_2_][OTf] was introduced in 10 mol% and trialkylsilylenol ethers were converted into trialkylhydrosilanes under mild conditions (1 bar of H_2_ at 50°C, 8 h in toluene). The release of a ketone is the driving force of the reaction and no further hydrosilylation of the carbonyl group took place. The key step in this reaction would involve a [Ru]─H species that can readily transfer its hydride to the silicon atom. This was confirmed experimentally in a stoichiometric control experiment under H_2_ (1 bar) between [Ru(H)Cl(dppe)_2_] and Me_3_SiOTf (1 eq) which afforded Me_3_SiH and [Ru(H_2_)Cl(dppe)_2_][OTf] almost quantitatively. A mechanism was suggested on the basis of these observations [64].

### 6.2 From Halosilanes and Silyl Triflates

**SCHEME 14:**
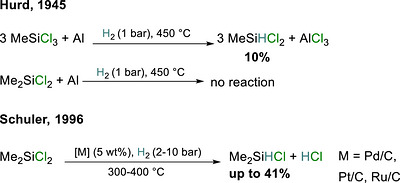
Heterogenous hydrogenolysis of chlorosilanes to hydrosilanes.

In 1945, Hurd demonstrated the formation of Si─H bonds during the reaction of an alkylchlorosilane with H_2_ (hydrogenolysis) or HCl by passing the reacting gas over finely divided aluminium(0) or zinc(0) at an elevated temperature (450°C) (Scheme 14) [65]. The reaction is not catalytic in metal and aluminium chloride is formed while yields of alkylhydrosilane are quite low (4 to 10%). Attempts to hydrogenate Me_2_SiCl_2_ were unsuccessful. The general absence of disilanes suggests that the reduction of chlorosilane to a silylanion intermediate is very unlikely. It is assumed that the formation of Si−H would result from the reaction of a transient aluminium hydride with the corresponding chlorosilane, as presented above (*vide supra* 3.2). The first metal catalysed process for the formation of hydrosilanes dates back to 1996. In this work, Schuler used palladium(0), platinum(0) and ruthenium(0) metal catalysts (5 wt%) supported on activated carbon to convert chlorosilanes to hydrosilanes [66]. Hydrogenolysis of Me_2_SiCl_2_ at 400°C afforded Me_2_SiHCl in 41% yield using Pt(0) catalyst but under these harsh conditions the Me_2_SiHCl compound partially disproportionates into MeSiHCl_2_ and Me_3_SiCl.

To avoid these drastic conditions and the associated selectivity issues, the use of a base is necessary to facilitate the heterolytic splitting of H_2_. Indeed, the reduction potential of H_2_ depends on the pH of the solution and can therefore be modulated by the pKa of a base in the medium. This strategy can make the hydrogenolysis reaction thermodynamically favourable even at room temperature. In addition, (noble) metal complexes can split dihydrogen in the presence of a base to generate metal hydrides.

**SCHEME 15:**
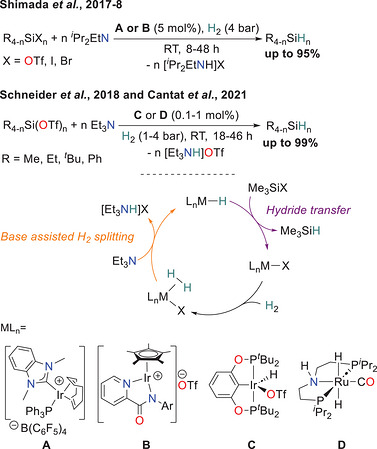
Catalytic hydrogenolysis of (pseudo)halosilanes to hydrosilanes.

In this context, the groups of Shimada, Schneider and Cantat [67, 68, 69, 70], reported the catalytic hydrogenolysis of Me_3_SiX compounds (X = OTf, I, Br) with the iridium(I or III) complexes **A**, **B,** or **C** or the ruthenium(II) complex **D**, in presence of the base *^i^* Pr_2_EtN or Et_3_N (Scheme 15), some of which have already been reviewed [71]. The nature of the (pseudo)halide X is crucial in these reactions. The transformation Me_3_SiX → Me_3_SiH with X = OTf or I is easy and proceeds under mild conditions (RT or at 60°C, 1–4 bar of H_2_) in high yields (up to 95%). In contrast, a maximum yield of 11% in Me_3_SiH is obtained from Me_3_SiBr while no reaction took place with Me_3_SiCl. The order of reactivity is as follows: OTf > I > Br > Cl. This reaction is also very sensitive to the steric hindrance on the silicon atom and the hydrogenolysis of Me_3_SiOTf and *^t^* BuMe_2_SiOTf gave the corresponding hydrosilanes in 93 and 6% yields respectively. A similar mechanism was proposed by the three groups and would rely on a base‐assisted cleavage of coordinated H_2_ with formation of a metal hydride. Subsequent hydride transfer can occur from the reactive metal complex to the silicon halide to produce the hydrosilane. This hydride transfer would follow a SN_2_‐type mechanism, comparable with previous observations of aluminium hydride reactivity (*vide supra* 3.2). Notably, in such a mechanism, the oxidation state of the metal catalysts would not change during the catalytic process.

**SCHEME 16:**
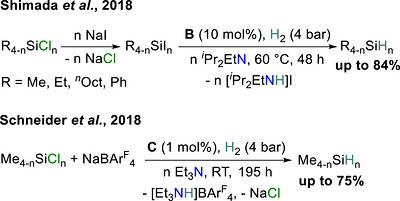
Catalytic hydrogenolysis of chlorosilanes in presence of chloride abstractors.

As chlorosilanes are readily available, they are the most appealing precursors to produce hydrosilanes. The hydrogenolysis of halosilanes to hydrosilanes is thermodynamically unfavourable, and in particular with the chlorosilanes where a bond dissociation energy difference of *ca*. 30 kcal.mol^−1^ between the Si─H and Si─Cl bonds must be overcome. The reactivity of chlorosilanes, as reported by Shimada and Schneider, can be circumvented by the stoichiometric use of chloride abstractors. Beppu et al., in a two‐step procedure [68], first added NaI to the chlorosilane to form in situ the corresponding iodosilane. Then, the catalytic hydrogenolysis by the iridium(III) complex **B** in the presence of the *^i^* Pr_2_EtN base was performed in a THF‐benzene mixture (Scheme 16). Thus, they obtained a variety of hydrosilanes in good yields (up to 84%) under mild conditions (60°C, 4 bar of H_2_). This halogen exchange also favoured formation of interesting mixed hydrochlorosilanes. Indeed, the reaction of di‐ and trichlorosilanes with NaI (1 eq) gave the corresponding iodochlorosilanes, which after hydrogenolysis led to the hydrochlorosilanes (Me_2_SiHCl, Ph_2_SiHCl, PhSiHCl_2_ or (*^n^* Hexyl)MeSiHCl) in yields varying from 50% to 70%.

The group of Schneider chose NaBAr^F^_4_ as the chloride abstractor and the reaction was carried out in one step in fluorobenzene [69]. The presence of the Et_3_N base is mandatory and the yields in hydrosilanes are moderate (37−75%). For example, Me_3_SiH was generated in 51% yield after 10 days at room temperature. In that case, the hydrogenolysis of Me_2_SiCl_2_ with NaBAr^F^_4_ (1 eq) leads to Me_2_SiH_2_ as the main product and not to the expected Me_2_SiHCl species. The higher reactivity of the ruthenium(II) hydride complex compared to the iridium(III) hydride species [68] may explain this difference in selectivity.

**SCHEME 17:**
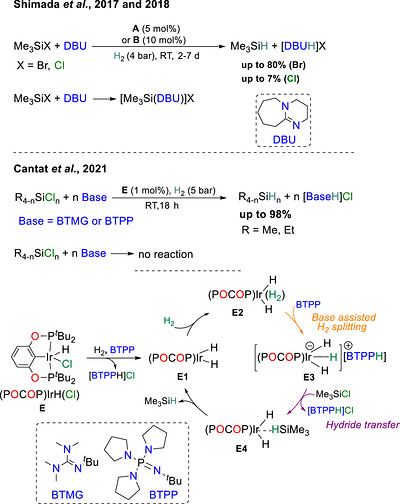
Catalytic hydrogenolysis of chlorosilanes using organic superbases.

A second strategy relies on the careful choice of the base to avoid abstraction of chlorides (Scheme 17). Amine bases (*^i^* Pr_2_EtN, Et_3_N) are too weak to induce the hydrogenolysis of chlorosilanes [69], so a stronger base is thermodynamically required. Because strong anionic bases such as hydroxide and alkoxide are not compatible with halosilanes, neutral bases have been considered. The choice of the amidine DBU (1,8‐diazabicyclo[5,4,0]undeca‐7‐ene), of higher pKa (24 vs. 19 for Et_3_N) proved beneficial [69]. With DBU (1.1 eq) and a 5 mol% catalytic charge of Ir(I) complex **A**, the hydrogenolysis of bromo‐ and chlorosilanes in toluene under 4 bar of H_2_ proceeds with 80 and 7% yields, respectively, but with long reaction time (7 days).

Improved hydrogenolysis of chlorosilanes under mild conditions (1‐5 bar of H_2_, RT) was carried out by the group of Cantat [72], using the iridium(III) pincer **E** (1 mol%) in presence of a guanidine or phosphazene bases (BTMG = 2‐*^t^* Bu‐1,1,3,3‐tetramethylguanidine; BTPP = *^t^* Bu‐imino)tri(pyrrolidino)phosphorane) (Scheme 17). The influences of steric congestion and base strength on hydrosilane yields were rationalised. The base must be sufficiently strong (pKa in MeCN >24) to induce reaction, but a high steric hindrance is also crucial to avoid the formation of a silylated adduct, [Me_3_SiBase]Cl, which is detrimental to the hydrogenolysis pathway. With bulky superbases such as guanidines or phosphazenes, the silylated adduct is not observed and the hydrogenolysis of chlorosilanes proceeds efficiently in dichloromethane. In the presence of BTPP or BTMG, excellent yields of hydrosilanes (up to 98%) are obtained. Monohydrogenolysis of dichlorosilanes was also carried out using one equivalent of the base, but it was found that when changing the class of silanes, the reaction must be re‐optimised. As an example, the most efficient base for the hydrogenolysis of Me_2_SiCl_2_ was found to be MeTBD (7‐Methyl‐1,5,7‐triazabicyclo(4.4.0)dec‐5‐ene) which gives Me_2_SiHCl in 54% yield. According to the stoichiometric experiments, the active species would be an anionic trihydride species [Ir(*^t^* ^Bu^POCOP)H_3_][BTPPH]. Indeed, reaction of this reactive complex with Me_3_SiCl gave Me_3_SiH very rapidly and quantitatively. A mechanism has been proposed which involves this species as drawn in Scheme 17.

**SCHEME 18:**
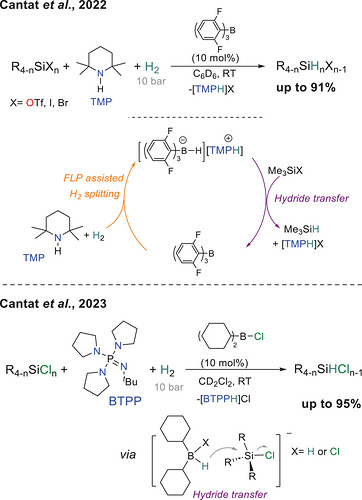
Metal‐free catalytic hydrogenolysis of silyl triflates and halosilanes.

All catalytic systems described so far for the hydrogenolysis of halosilanes used rare and expensive noble metals. Capitalising on the above work, and in particular on the fact that the oxidation state of the metal does not change during catalysis (*vide supra* 6.2), our group reported the first metal‐free hydrogenolysis of silyl triflates and halosilanes using organoborane catalysts (Scheme 18) [73]. This discovery is based on the chemistry of frustrated Lewis pairs (FLP) and their remarkable ability to activate H_2_. A number of triarylboranes have been evaluated, B(2,6‐F_2_─C_6_H_3_)_3_ or B(2‐F─C_6_H_4_)_3_ combined with the hindered tetramethylpiperidine (TMP) were found to be efficient catalysts to produce Me_3_SiH from Me_3_SiX (X = OTf, I, Br) in high yields (up to 91%). Although these results represent a further step toward a virtuous synthesis of hydrosilanes, fluoroarylboranes, regardless of the nature of the base, were found to be ineffective in converting chlorosilanes to hydrosilane. Me_3_SiCl being the least reactive of the (pseudo)halosilanes, the idea came to generate a more reactive borohydride by splitting H_2_ with a weakly acidic borane (Cy_2_BCl) in the presence of the strong phosphazene base BTPP, inspired by Krempner's work on inverse FLPs [74]. This particular combination showed indeed the formation of Me_3_SiH (95%) from Me_3_SiCl after 48 h in dichloromethane after optimization [75]. The associated mechanistic study suggests the formation of highly reactive borohydride species of the type [Cy_2_BHX]^‒^, (X = H or Cl). These new metal‐free catalytic system are thus effective at room temperature in CD_2_Cl_2_ or benzene but requires a higher catalytic loading and H_2_ pressure than noble metals (10 mol% of borane and 10 bar of H_2_). Nevertheless, these methods are competitive with that using iridium or ruthenium catalysts in terms of scope, yields and reaction time.

Despite these recent synthetic efforts towards hydrosilanes, these catalytic hydrogenolysis procedures are still limited to a narrow scope of halosilanes. Thus, more efficient and general catalysts will have to be designed to overcome this shortcoming. Moreover, in these processes, the bases are used in stoichiometric amounts. In order to have a more virtuous process, the base will have to be recycled. Finally, no heterogeneous catalyst has been described for this reaction where a base is involved and such a catalyst could have advantages over homogeneous catalysts in terms of recyclability for example.

## 7 Energy Efficiency of the Different Hydrosilane Synthetic Routes

All reactions reported in this review describe the conversion of (pseudo)halosilanes or some alkoxysilanes to hydrosilanes with the use of electrochemistry (electron and proton sources) or chemical reductants such as metal hydrides or molecular H_2_ (Table 1). Access to elemental silicon, currently produced from SiO_2_ and coal at about 2000°C, and the conditions of the industrial Müller–Rochow process to produce chlorosilanes from Si(0) are no longer expected to be the main route for the production of hydrosilanes. Especially, if they are to be used on an industrial scale for the valorisation of oxygenated feedstocks. Metal hydrides of alkali metals (LiH, NaH), alkaline rare earths (MgH_2_, CaH_2_) or Grignard reagents are powerful reducing agents and are all obtained from the corresponding elemental metal (Li, Na, Mg or Ca) by reaction with H_2_ (or RX) at high temperature [76]. Therefore, a potential of at least –2.4 vs. SCE is employed and the energy efficiency is very low.

**TABLE 1:** Summary of the hydrosilane synthetic methods reported in this review.

Hydrosilane product	Substrate	Reagents / method	References
R_3_SiH (R = alkyl)	R_3_SiCl R_3_SiOMe and R_3_SiCl (silyl enol ethers) R_3_SiX (X = OTf, I or Br) (X = OTf)	NaH (HMPT, r.t.) MgH_2_ (titanium cat., DME, 85°C) NaBH_4_ (MeCN, r.t.) Na_2_[DBA]H_2_ (THF, r.t.) *^n^* Bu_3_SnH (LB cat., Ether, r.t.) RMgBr ([L_2_MCl_2_] cat., Et_2_O, r.t.) H_2_, Et_3_N, NaBAr^F^_4_ ([**C**] cat., C_6_D_6_, r.t.) H_2_, BTMG or BTPP ([**E**] cat., CD_2_Cl_2_, r.t.) H_2_, BTPP (Cy_2_BCl cat., CD_2_Cl_2_, r.t.) NaH (mineral oil, >250°C) *inc. siloxane* LiAlH_4_ (DME, 70°C) HBpin (Yttrium cat., C_6_D_6_, 100°C) NaBH_4_ + EtBr (HMPA cat.,^a^ C_6_D_6_, r.t.) BH_3_(THF) (HMPA, THF, r.t.) H_2_ ([Ru] cat., DCE, 50°C) H_2_, *^i^* Pr_2_EtN ([**A** or **B**] cat., C_6_D_6_, r.t.) H_2_, Et_3_N (B(2,6‐F_2_─C_6_H_3_)_3_ cat., C_6_D_6_, r.t.) H_2_, Et_3_N ([**C** or **D**] cat., C_6_D_6_, r.t.)	[31] [34] [41] [42] [46, 47, 48] [49, 50, 51] [69] [71] [75] [28, 29, 30] [38] [43] [44] [44] [64] [67, 68] [73] [69, 70]
R_3_SiH (R = aryl)	R_3_SiCl	*^t^* Bu_2_AlH (*^n^* hexane, r.t.) 2 e^−^, PhOH (Hg or Pt cathode, DME, r.t.) Sacrificial Al cathode (DME, – 6°C) then hydrolysis H_2_, BTPP (Cy_2_BCl cat., CD_2_Cl_2_, r.t.)	[39] [62] [63] [75]
R_2_SiHCl	R_2_SiCl_2_	LiH (phosphonium cat., diglyme, 120°C) CaH_2_ (neat, 320°C) Na_2_[DBA]H_2_ (THF, r.t.) H_2_ (Pd/C, Pt/C or Ru/C cat., 300–400°C) H_2_, BTPP (Cy_2_BCl cat., CD_2_Cl_2_, r.t.)	[32] [33] [42] [66] [75]
RSiHCl_2_ RSiH_3_ (not pure)	RSiCl_3_	LiH (phosphonium cat., diglyme, 120°C) NaH (300°C)	[32] [30]
R_2_SiH_2_	R_2_SiCl_2_ R_2_Si(OMe)_2_ R_2_Si(OTf)_2_	LiH (Dioxane, 100°C) NaH (HMPT, r.t.) LiAlH_4_ / NaAlH_4_ (Et_2_O, 0°C) LiBH_4_ (neat, r.t.) NaBH_4_ (MeCN, r.t.) *^n^* Bu_3_SnH (LB cat., Ether, r.t.) RMgBr ([L_2_MCl_2_] cat., Et_2_O, r.t.) HBpin (Yttrium cat., C_6_D_6_, 100°C) NaBH_4_ + EtBr (HMPA cat.,^a^ C_6_D_6_, r.t.) H_2_, Et_3_N ([**C**] cat., C_6_D_6_, r.t.) H_2_, Et_3_N (B(2,6‐F_2_─C_6_H_3_)_3_ cat., C_6_D_6_, r.t.)	[26] [31] [26] [40] [41] [46, 47, 48] [49, 50, 51] [43] [44] [69] [73]
RSiH_3_	RSiCl_3_ RSi(OMe)_3_	NaH (mineral oil, 250°C) NaBH_4_ (MeCN, r.t.) NaBH_4_ + EtBr (HMPA cat.,^a^ C_6_D_6_, r.t.) HBpin (Yttrium cat., C_6_D_6_, 100°C)	[27/30] [41] [44] [43]

^a^ *n*Oct_4_NBr as cocatalyst

Although aluminium hydrides, borohydrides and their neutral derivatives (alanes, boranes) as well as hydrostannanes have less negative redox potentials between –1.1 and –2.3 V compared to SCE, they are also generated by the reaction of metal hydrides such as LiH or NaH and their corresponding perhalogenated precursors [76]. Therefore, the energy efficiency of their production is still very low, and the atom economy is worse than the direct use of an alkali metal hydride for an overall hydrosilane synthesis.

**SCHEME 19:**
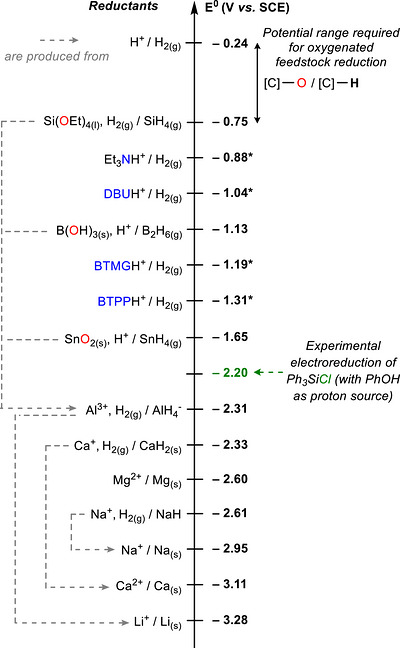
Redox potential scale of the reductants converted vs. SCE if required [9, 77]. *These redox potentials derived from the pKa [78, 79] values of the BaseH^+^/Base couple following a previously reported method [80].

Electrochemistry has great potential for the synthesis of renewable hydrosilanes. However, preliminary studies have shown that the reduction of chlorosilanes to hydrosilanes requires very negative potentials (at least –2.2 V vs. SCE) while the theoretical reduction potential should be *c.a. –*0.75 V vs. SCE. It means that specific catalysts are required to overcome this overpotential of *∼*1.5 V for this transformation (Scheme 19).

**SCHEME 20:**
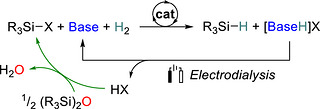
Electrodialysis strategy for base recycling. Cat represents a catalyst.

Currently, dihydrogen is mainly obtained by steam reforming of methane [81]. But emerging processes based on electro‐ and photocatalytic splitting of water with renewable energies offer the prospect of significant and sustainable H_2_ production. Dihydrogen is a poor reductant with a redox potential E^0^ = –0.24 V vs. SCE (at pH = 0). However, its reducing properties can be modulated to overcome the thermodynamic limitations. The bases (amines, amidines, guanidines and phosphazenes) used in the previous studies [67, 68, 69, 70, 72, 73] decrease the H_2_ reduction potential in the range of –0.88 to –1.31 V vs. SCE, making these systems more efficient than metal hydrides. However, if a base is used in a stoichiometric amount, its recycling must be considered and then factored into the overall energetic cost of the reaction. The most appealing approach to recycle the base would be electrodialysis (Scheme 20). This membrane‐based process was shown to be effective for the synthesis of ammonia and hydrogen chloride from ammonium chloride in water [82]. This process should then be transposed to bases of interest such as triethylamine, guanidine BTMG or phosphazene BTPP. However, it should be noted that some of these bases are not compatible with water and that an organic solvent must be used. Successful regeneration of the base would lead to the formation of the acid HX as a by‐product which could then be used for the dehydration of siloxanes necessary to close the silicon cycle (*vide supra* Scheme 1).

## 8 Concluding Remarks

This review summarises the different ways to synthesise hydrosilanes by electrochemical or chemical methods. The oldest ones, involving high temperatures or highly reactive reagents, are described and still used industrially. More recent developments aim at less energy consuming preparation methods by favouring milder conditions and by using H_2_ as reductant in combination with metallic (or organic) catalysts. Improvements are still to be expected, both in terms of energy efficiency and the nature of the reagents, their abundance and recyclability.

Future research for the production of hydrosilanes should focus on two objectives. The first is to improve the energy efficiency of the Müller–Rochow industrial process by accessing chlorosilanes in a more sustainable manner. The second will be the easy recycling of Si–O bonds from siloxanes into value‐added compounds involving Si–X and Si–H derivatives. This will be crucial especially in the case of using hydrosilanes for reductive depolymerisation of oxygenated bio‐based materials (lignin, cellulose) or recycling of waste plastics (polyesters, polycarbonates) to provide compounds of interest to the chemical industry.

For the transformation of Si–O to Si–H, the hydrogen source must be abundant and sustainable. Dihydrogen or the electron/proton couple will certainly be the main sources, provided that they come from renewable sources and energies. To avoid energy‐intensive processes, catalysis will be necessary. The search for stable and efficient non‐noble metal catalysts that can both activate dihydrogen and promote cleavage of strong Si–O or Si–Cl bonds is a challenge. From a mechanistic point of view, the main reactivities explored so far do not rely on any change of oxidation state for the catalyst and are based on SN_2_‐type hydride transfers. Other activation modes of these Si–X bonds should also be considered for catalysis such as oxidative addition or hydride transfer based on SNi‐type mechanism. In addition, no electrocatalyst has yet been developed for the synthesis of hydrosilanes. Therefore, future advances in this field must address these challenges.

## Conflicts of Interest

The authors declare no conflicts of interest.

## Data Availability

Data sharing not applicable to this article as no datasets were generated or analysed during the current study.
